# Fitness Costs of Mutations at the HIV-1 Capsid Hexamerization Interface

**DOI:** 10.1371/journal.pone.0066065

**Published:** 2013-06-13

**Authors:** Siriphan Manocheewa, J. Victor Swain, Erinn Lanxon-Cookson, Morgane Rolland, James I. Mullins

**Affiliations:** Department of Microbiology, University of Washington, Seattle, Washington, United States of America; University of Hawaii Manoa, United States of America

## Abstract

The recently available x-ray crystal structure of HIV-1 capsid hexamers has provided insight into the molecular interactions crucial for the virus’s mature capsid formation. Amino acid changes at these interaction points are likely to have a strong impact on capsid functionality and, hence, viral infectivity and replication fitness. To test this hypothesis, we introduced the most frequently observed single amino acid substitution at 30 sites: 12 at the capsid hexamerization interface and 18 at non-interface sites. Mutations at the interface sites were more likely to be lethal (Fisher’s exact test p = 0.027) and had greater negative impact on viral replication fitness (Wilcoxon rank sum test p = 0.040). Among the interface mutations studied, those located in the cluster of hydrophobic contacts at NTD-NTD interface and those that disrupted NTD-CTD inter-domain helix capping hydrogen bonds were the most detrimental, indicating that these interactions are particularly important for maintaining capsid structure and/or function. These functionally constrained sites provide potential targets for novel HIV drug development and vaccine immunogen design.

## Introduction

Human immunodeficiency virus type 1 (HIV-1) is an enveloped retrovirus with a protein shell, or capsid, enclosing the viral genome and its associated enzymes. The immature capsid is formed inside a host cell as a spherical shaped particle made up of Gag polyproteins. Upon budding and release from the host cell, the Gag polyproteins are cleaved by the viral protease, resulting in separation of CA from other functional domains of Gag. The CA units then rearrange to form a more condensed, cone-shaped mature capsid. This process is called maturation [1] and is necessary for successful production of infectious virions. The mature HIV-1 capsid is organized like a fullerene cone, and is made up of approximately 250 hexamer and 12 pentamer units of CA [2], [3]. Each CA protein consists of two independently folded domains – the amino-terminal domain (NTD) and the carboxyl-terminal domain (CTD). The two domains are joined by a short unfolded region called the flexible linker [4]. A recently solved high-resolution crystal structure of the capsid hexamer revealed two main interfaces between CA subunits within the hexamer – the NTD-NTD and NTD-CTD interfaces [5].

Amino acid changes at these interface sites have the potential to interrupt CA-CA interactions and hence destabilize the hexamer structure. Such mutations would also be expected to have a negative impact on viral replication, as previous studies showed that mutations that affected capsid morphology and stability also abolished viral infectivity [6]–[9]. Indeed, some mutations at several interface sites were shown to result in less-, or in some cases, non-infectious virus, while others did not have the same negative effects, suggesting that side-chain interactions had different effects on viral infectivity [7].

Identification of critical residues or side-chain interactions may facilitate the development of novel anti-HIV therapies targeting the capsid [10], including immunogens for cytotoxic T lymphocyte (CTL) based HIV vaccines. CTL responses are crucial for control of viral replication during infection [11], [12]. However, while CTL responses are extensive, their effectiveness is mitigated by immune escape mutations, which allow the virus to evade immune responses and continue to replicate [13], [14]. Focusing immune responses on the functionally constrained regions of the viral proteome can potentially alleviate this problem, as immune escape mutations may be expected to impair viral replication and reduce viral spread [15], [16]. This information would also be useful for development of novel anti-HIV drugs targeting the capsid [10].

A structural characterization of the CA-CA interface within capsid hexameric units was presented in 2009 [5], but the effects of mutations at many of these interface sites have not been investigated. Importantly, most of the previous work utilized alanine-scanning mutagenesis or other non-conservative amino acid changes to study alterations in CA production, capsid formation and viral infectivity. While such approaches are suitable for studying a functional or structural role of specific residues within a protein or protein complex, they did not take into account the likelihood of the observed mutations at a given residue during HIV evolution. Such information is useful when considering potential sites for drug target or vaccine immunogens and plausible drug/immune escape mutations. To gain such information, we introduced the most frequently observed mutation in natural infections at 12 interface sites and 18 non-interface sites and studied their impact on viral fitness. Our results showed that commonly observed mutations at interface sites were more likely to have deleterious effects on viral replication than mutations at non-interface sites. These results support the potential of CA-CA interfaces as drug targets and for inclusion of these sites in vaccine immunogens.

## Materials and Methods

### Mutation Sites

The capsid hexamerization interface site is defined as any amino acid residue that forms at least one non-bonded contact with a residue from another CA subunit within the same capsid hexamer. The number and type of non-bonded contacts was obtained from the PDBsum [17] using the x-ray crystal structure of the capsid hexameric unit (PDB code 3H4E). Twelve sites were selected for mutagenesis, six were located in the NTD-NTD interface and the other six were in the NTD-CTD interface. Eighteen non-interface sites were selected for mutagenesis to include sites that were located throughout CA, at which no specific functional roles have been reported and which were differentially conserved.

### Amino Acid Substitution

At each selected interface and non-interface site we introduced a mutation in the COTM prototype (see below): usually the most frequently found amino acid was changed to the second most frequently observed one based on a dataset of circulating sequences. The amino acid frequency at each residue was determined as follows. First, all HIV-1 group M *gag* sequences were downloaded from the February 2010 HIV sequence database at the Los Alamos National Laboratory (HIVDB), without regard for duration of infection. The sequences were then screened to carefully exclude all but one sequence per subject or linked pairs of subjects. In addition, only full-length *gag* sequences with a proper start codon, without an early stop codon or frame-shift mutation, were used for the alignment. The multiple sequence alignment was prepared using the MUSCLE program and then manually edited using Mesquite [18]. The database frequency of each amino acid at each site was then calculated using a perl script (http://indra.mullins.microbiol.washington.edu/perlscript/docs/CountAAFreq.html). In addition, alanine mutations were introduced at two interface sites, T186 and F301.

### COTM CA Sequence

The impacts of mutations on viral fitness were estimated in the context of the computationally derived Center-Of-Tree group M (COTM) CA sequence. The COTM sequence corresponds to the minimum evolutionary distance to all circulating group M viral sequences while still residing on an evolutionary path. It retains co-varying residues but was not biased toward outlier sequences [15], [19]. The phylogenetic tree and the COTM CA sequence were determined, as part of the COTM Gag sequence, based on the selected group M viral sequences obtained from the HIVDB using DIVEIN [20] (http://indra.mullins.microbiol.washington.edu/DIVEIN/diver.html).

### Molecular Clone Construction

#### Plasmid vector construction

Two restriction sites, *Sfi*I and *Bst*EII, flanking the 5′ and 3′ ends of the *gag-ca* region, were introduced into pNL4-3 molecular clones using synonymous site changes, with or without a *vif* tag – a set of six additional synonymous mutations located in *vif* gene (kindly provided by Dr. Eric J. Arts, Case Western Reserve University) – using synthetic oligonucleotide primers with the QuikChange XL II Site-directed mutagenesis kit (Agilent). These nucleotide differences were used to differentiate the prototype and mutant strains in competitive fitness assays. The same restriction sites were also engineered using synonymous site changes into the 222H plasmid, containing COTM *gag* (kindly provided by Dr. Barbara Felber, NCI). Primer sequences are listed in Table S1.

The COTM-CA coding fragment was isolated from the modified 222H plasmid by *Sfi*I and *Bst*EII double digestion, gel separation and band extraction. The pNL4-3 vectors were prepared similarly. The COTM-CA coding fragment was inserted into the pNL4-3 vectors using T4 ligase (Invitrogen). We designated the chimeric plasmid with a *vif* tag ‘COTM-CA-vifB’ and the original chimeric plasmid ‘CotM-CA-vifA’. All amino acid altering mutations were then introduced into the chimeric CotM-CA-vifA plasmid.

The engineered plasmids were transformed and propagated in electrocompetent DH10B *E. coli* cells using the ElectroMax kit (Invitrogen) following the manufacturer’s protocol. Plasmid DNA was prepared using the HiSpeed Plasmid Midi Kit (Qiagen). The entire plasmid was sequenced to confirm the presence of the newly engineered restriction sites and the absence of extraneous mutations that could have arisen during the mutagenesis process.

Individual mutations were introduced into the chimeric pNL4-3-COTM-CA plasmid using the QuikChange II Kit and plasmid DNA was prepared using the HiSpeed Plasmid Midi Kit with an additional endotoxin removal step. The DNA sequence of the entire HIV-1 genome in mutated plasmids was determined to confirm the presence of the desired mutations and the absence of additional mutations.

### Generation of HIV-1 Chimeric Viruses

Viral stocks were generated by transfecting HEK 293T-17 cells with 1 ug of the chimeric plasmid using the FuGENE6 DNA transfection reagent (Roche). Cell-free supernatants were harvested 48 hours post-transfection, filtered through a 0.22 um filter, and stored in 250 ul aliquots at -80C until use. p24 production in transfection supernatants was determined using an in-house double-antibody sandwich ELISA specific to the HIV-1 p24 antigen [21]. The viral titer for each stock was calculated in quadruplicate as the 50% tissue culture infectious dose (TCID_50_) using CEMx174 cells and following the Reed and Muench method [22]. Positive wells were scored based on the presence of cytopathogenic effects (CPE). The titer was considered undetectable for mutants showing no CPE. For confirmation, transfection and TCID_50_ determinations were repeated for mutants showing no CPE.

### Relative Fitness Assays

The COTM-CA with the *vifB* tag was considered the prototype virus. The replication fitness of all other viruses was estimated in relation to this prototype.

#### Viral growth competition (dual-infection) assay

All competition experiments were done in the CEMx174 cell line cultured in RPMI-1640 medium (Sigma) supplemented with 10% fetal bovine serum (FBS) (Sigma) and 1% glucose. Cultures were incubated at 37°C with a 5% CO_2_ atmosphere. Experiments were done in triplicate at 10^5^ cells/well in a 48 well plate using a viral multiplicity of infection (MOI) of 0.005. Viral inocula were prepared with the mutant and reference viruses at a ratio of 1∶1 based on TCID_50_. The inoculum volume was 0.5 ml and the total volume of viral-cell cultures were 1 ml per well. After 16 hours, 0.75 ml of the culture supernatant was removed and replaced with warm complete RPMI-1640. Cultures were spun for 5′ at 228×g, and then 0.75 ml of the culture supernatant was again replaced. On days 0, 3, 5, 7 and 9, 0.5 ml of culture supernatant was replaced and the removed supernatant transferred to a sterile 1.8 ml microcentrifuge tube. Supernatants were centrifuged for 5′ at 228×g and cell free fluids were stored at −80°C in 200 ul aliquots.

#### Viral growth (mono-infection) assay

Viral-cell cultures were set up as described above using an MOI of 0.005. For repeated analysis of mutants with undetectable infectious titer, 0.5 ml of viral stock was used as inoculum.

#### Nucleic acid preparation and amplification

Viral RNA was extracted from 200 ul cell-free supernatants by an ion exchange membrane technology on a QIAxtractor robot following the manufacturer’s protocol (Qiagen). RNA was stored at −80°C prior to cDNA synthesis. One 25 ul cDNA synthesis reaction consisted of master mix 1 (final concentration: 0.5 mM dNTPs, 0.5 uM RT-2 primer), master mix 2 (final concentration: 1X first strand buffer, 0.005 M DTT, 10U/ul Superscript III reverse transcriptase (Invitrogen), 2U/ul RNase Inhibitor (Invitrogen), and 10 ul RNA. Four ul of master mix 1 was transferred to each reaction well in a 96 well PCR plate. Ten ul of RNA was added to each reaction well and incubated at 60°C for 5 min. The reaction was then held at 4°C until master mix 2 was prepared. Ten ul of master mix 2 was added to each reaction well and the mixture incubated for 90 minutes at 50°C. Reverse transcriptase was inactivated by incubating the reaction for 15 minutes at 70°C and the reaction was then held at 4°C until real-time PCR quantitation.

#### Real-time quantitative PCR (qPCR)

Viral RNA was quantified to determine the growth kinetics of each virus. All mutant viruses were generated using pNL4-3 with a vif tag (vifB) and were competed against the vifA reference virus. Viral RNA from the two viral strains was distinguished based on the presence or the absence of the vif tag mutations [23]. Real-time qPCR was done using an ABI 7300 Real-Time PCR System (Life Technologies). One 25 ul PCR reaction contained 12.5 ul TaqMan® Gene Expression Master Mix (Life Technologies), 5 uM probe, 20 uM of the forward and reverse primers [24], and 1 ul of cDNA. PCR cycling parameters were 50°C for 2 min, 95°C for 10 min, followed by 40 cycles at 95°C for 15 sec and 60°C for 1 min. A standard curve was generated using pNL4-3 diluted serially from 3x10^7^ to 300 copies/ul run in triplicate wells. The copy number of cDNA and corresponding Ct values was used to extrapolate cDNA copy from each sample. Each sample was run in duplicate wells.

#### Estimating relative fitness

All relative fitness estimations were done using the vFitness web tool [25]. The death rate of prototype and mutant viruses were set at 0.5. The least squares method was used to estimate fitness parameters from multiple data points. Data from the time points within the exponential growth phase were used for the relative fitness (1+s) calculation [26]. Thus, the viral cDNA copy number measurements from day 3 and 5 samples were used when the COTM virus was competed against the NL4-3, while the copy number from day 3, 5 and 7 were used for competitions between COTM vifA and the COTM vifB mutants.

## Results

### Chimeric HIV-1 COTM-CA Viruses

**Figure 1:**
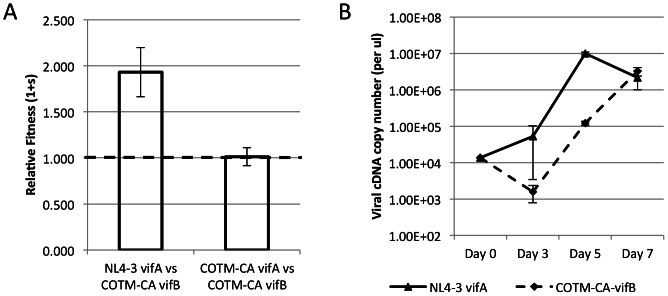
Relative fitness and growth kinetics of NL4-3 and COTM-CA viruses. **A**) COTM-CA vifB, containing six synonymous site changes in vif, relative to COTM-CA vifA, was competed against COTM-CA vifA and the NL4-3 prototype strain. Relative fitness values shown are the average from two experiments done in triplicate. The error bars represent the 95% confidence interval. The dotted line represents neutral fitness. **B**) Viral growth over seven days in cell culture. Values shown are the average from one experiment done in triplicate. The error bar represents the standard deviation.

The COTM-*gag* sequence was computationally derived to represent the central point of group M viral sequence diversity [19], [27]. 1,019 full-length *gag* sequences, representing all group-M HIV-1 subtypes and circulating recombinant forms (CRFs), were obtained from the HIVDB: ∼40% of the sequences were subtype B, and ∼40% were subtype C, all other subtypes together composed ∼10%, and the remaining 10% corresponded to CRFs (Table S2). As the COTM sequence differed from known natural isolates or lab strains, we first confirmed that both COTM-CA-vifA and COTM-CA-vifB viruses were infectious in the CEMx174 T-cell line. We then competed the COTM-CA-vifA against the COTM-CA-vifB virus and showed that the six synonymous mutations in *vif* did not have an impact on viral replication fitness (Figure 1A). The COTM-CA-vifB virus was also competed against the NL4-3 virus, and was found to have a slower growth rate and hence lower relative fitness than the highly culture adapted NL4-3 (Figure 1A and 1B).

### Mutations and CA Sequence Conservation

The amino acid frequency at each site within CA was determined from the 1,019 codon-aligned sequences. CA sequences were relatively conserved across group M, with an average database frequency of the prototype amino acid of 0.94. Overall, capsid hexamerization interface sites were as conserved as other sites (Wilcoxon rank sum test p = 0.4317). However, when restricted to the sites included in this study, the interface sites tended to be slightly more conserved than non-interface sites but the difference did not reach statistical significance (Wilcoxon rank sum test p = 0.072) (Table S3).

Base on database frequency, the 30 amino acid sites included in this study can be categorized into three groups: 1) 18 sites whose mutation pattern was conserved across group M subtypes, 2) 8 sites whose subtype B mutation pattern is the opposite of subtype C, and 3) 4 sites whose mutation pattern in subtype B and subtype C are similar but different from that of other group M subtypes (Table S5). At all 30 sites, the most frequently found amino acid was changed to the second most frequently observed one. In addition, alanine mutations were introduced at two interface sites, T186 and F301.

### Impact of Single Amino Acid Changes on COTM-CA Viral Replication

**Figure 2:**
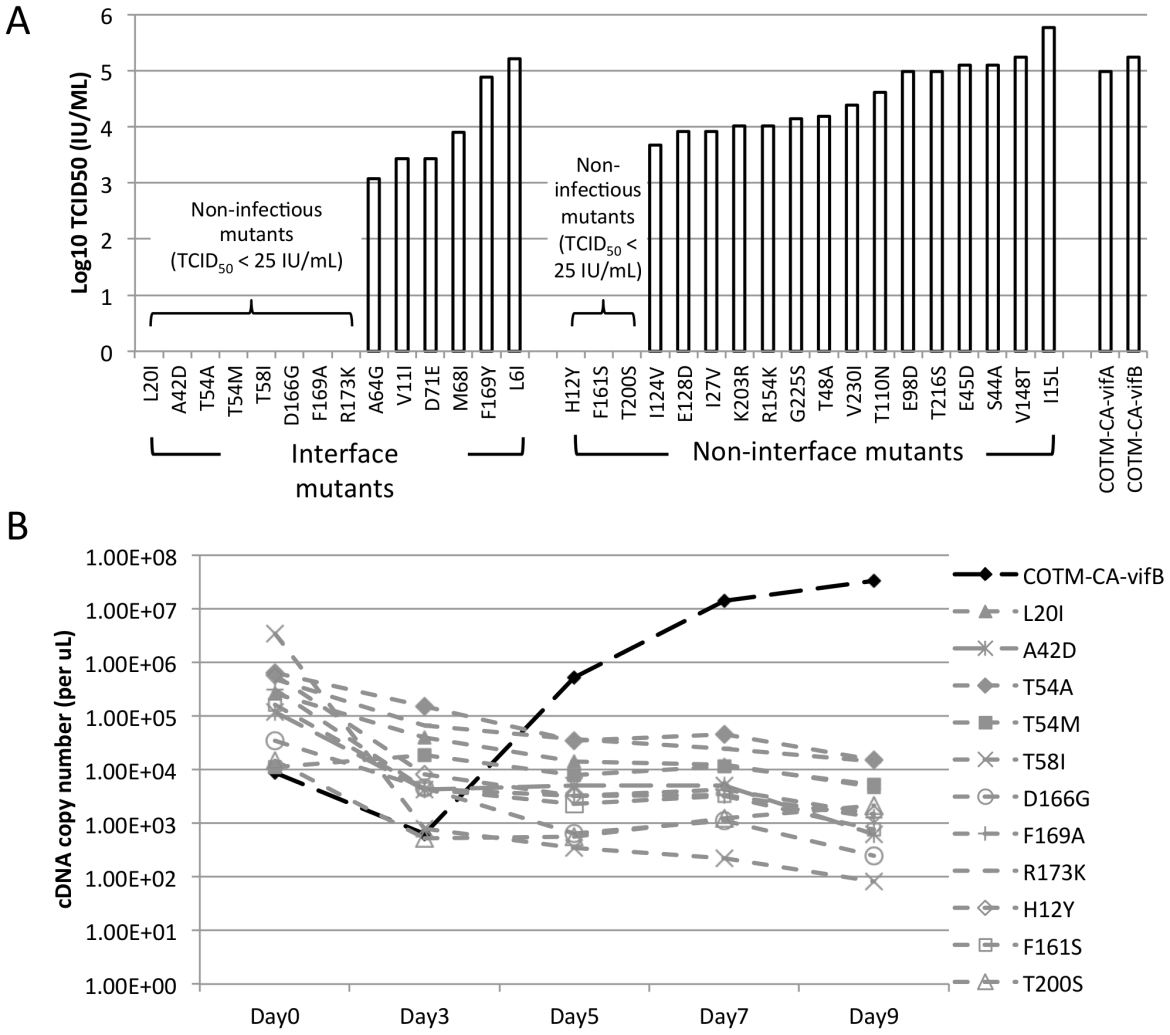
Growth kinetics of the prototype COTM-CA virus and its mutants in CEMx174 cells. **A**) Log_10_ TCID_50_ of viral stocks determined in CEMx174 cells. **B**) Decay of non-infectious mutants in cell culture (gray lines). These mutants showed no cytopathic effects when cultured in CEMx174. Values shown are the average from one experiment done in triplicate.

Thirty-two COTM-CA-vifA mutants were generated. CA production was detected in all culture transfected with mutant plasmids. Transfection with four mutant plasmids (T58I, D166G, F161S, and T200S) yielded 20 fold or lower levels of CA production when compared to the prototype plasmid (Figure S2). From each viral stock, viral RNA was extracted, DNase treated and sequenced to confirm that no spurious mutation arose during the transfection process. The infectious titer of each viral stock was then determined (Figure 2A). Eleven of these mutants did not show detectable cytopathogenic effects (CPE) or CA production in TCID_50_ assays, in contrast to the prototypes and the other twenty-one mutants, suggesting that these mutants could not replicate at detectable levels in CEMx174. We also infected 10^5^ CEMx174 cells with 0.5 ml of mutant viral stocks from the apparently defective mutants, along with 500 infectious units (IU) of COTM-CA-vifB and measured the amount of viral RNA from the culture supernatant on day 0, 3, 5, 7 and 9. For all eleven mutants, no growth was detected, although the mutant viral RNA remained above detectable levels (Figure 2B). Although we cannot completely rule out the possibility of the mutants replicating at very low levels, the mutant viral RNA detected is likely to be residual from the viral RNA in the inoculum as these levels steadily declined throughout the culture period (Figure 2B). Four out of these eleven non-infectious mutants also showed the lowest CA production in the 293T-transfected culture (Figure S2). These mutations may interfere with the CA expression and/or the release of viral particle from host cell.

**Figure 3:**
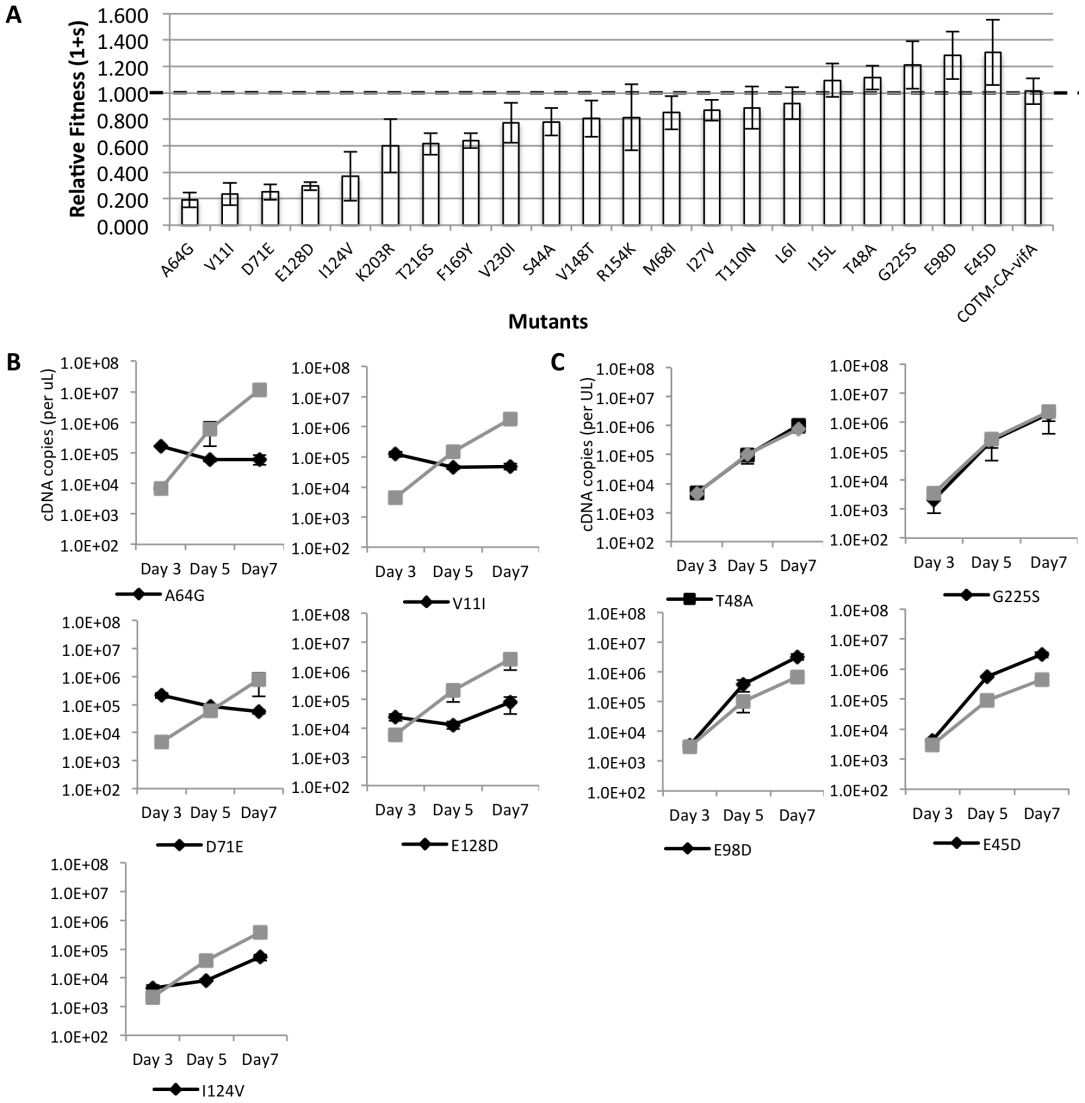
Viral replication fitness and growth kinetics of COTM-CA mutants in pairwise competition assays. **A**) Relative fitness of the 21 viable mutants in CEMx174 cells. Values shown are an average from two experiments, with three replicates each. Error bars represent 95% confidence intervals. The dotted line represents neutral fitness. **B**) Growth kinetics of the five mutants (black lines) with substantial lower fitness compared to the COTM-CA prototype virus (gray lines). **C**) Growth kinetics of the four mutants with higher replication fitness than the prototype. Values shown are the average from one selected experiment done in triplicate. Error bar represents the standard deviation.

Next, we assessed the relative fitness of each of the other twenty-one mutants by competing them against the prototype COTM-CA-vifB virus in dual growth competition assays. At each time point, the mutant and the prototype viral RNA were quantitated using specific primer-probe set for vifA and vifB sequence respectively. Viral replication fitness was calculated based on the change in prototype and mutant viral ratio over time (Figure S1). The mean relative fitness (1+s) of these mutants was spread over a wide range, from 0.21 to 1.31 (Table 1). The majority of the mutations resulted in decreased replication fitness, with 5: A64G (mean 1+s = 0.21), V11I (0.24) D71E (0.25), E128D (0.30), and I124V (0.37), having the largest fitness costs (Figure 3A). These five mutants exhibited a longer lag phase of viral production in competition experiments and slower growth kinetics (Figure 3B). Eight mutations: K203R, T216S, F169Y, V230I, S44A, V148T, M68I and I27V, resulted in varying but less severe fitness cost with mean 1+s ranges from 0.60 to 0.87. Three mutants: R154K, T110N, and L6I, also showed a trend of reduced fitness, although not significantly different from the prototype (95% CI p>0.05). Mutation I15L resulted in a trend toward higher replication fitness (95% CI p>0.05) while four mutants: T48A (mean 1+s = 1.12), G225S (1.21), E98D (1.28) and E45D (1.31), had a small but significantly faster growth rate than the prototype virus in all six replicates from two experiments (p<0.05) (Figure 3A and 3C).

**Table 1:** Amino acid database frequency, infectious titer and relative fitness of thirty-two COTM-CA mutations.

Location	Mutation	Amino acid frequency		Log_10_ TCID50	Relative Fitness (1+s)^a^
		Prototype	Mutated		
	Prototype			5.24	1.00
NTD-NTD	L6I	0.687	0.066	5.21	0.84
	V11I	0.939	0.037	3.43	0.24
	L20I	1.000	0.000	Undetectable	–
	A42D	1.000	0.000	Undetectable	–
	T54M	0.867	0.096	Undetectable	–
	T54A	0.867	0.005	Undetectable	–
	T58I	0.876	0.103	Undetectable	–
NTD-CTD	A64G	0.999	0.001	3.07	0.21
	M68I	0.981	0.013	3.9	0.85
	D71E	0.554	0.439	3.43	0.25
	D166G	0.998	0.002	Undetectable	–
	F169Y	0.538	0.456	4.89	0.64
	F169A	0.538	0.000	Undetectable	–
	R173K	0.999	0.001	Undetectable	–
Non-interface	H12Y	0.984	0.013	Undetectable	–
	I15L	0.617	0.320	5.77	1.10
	I27V	0.575	0.419	3.91	0.79
	S44A	0.965	0.029	5.1	0.78
	E45D	0.987	0.008	5.1	1.31
	T48A	0.984	0.006	4.18	1.12
	E98D	0.918	0.074	4.98	1.28
	T110N	0.861	0.118	4.62	0.89
	I124V	0.739	0.246	3.67	0.37
	E128D	0.601	0.390	3.91	0.30
	V148T	0.663	0.274	5.24	0.81
	R154K	0.555	0.438	4.02	0.82
	F161S	0.993	0.002	Undetectable	–
	T200S	0.842	0.091	Undetectable	–
	K203R	0.588	0.406	4.02	0.60
	T216S	0.976	0.009	4.98	0.62
	G225S	0.562	0.416	4.14	1.21
	V230I	0.925	0.066	4.38	0.77

a Values shown are the average from two experiments done in triplicate.

### No Relationship between Sequence Conservation and Fitness Effects

**Figure 4:**
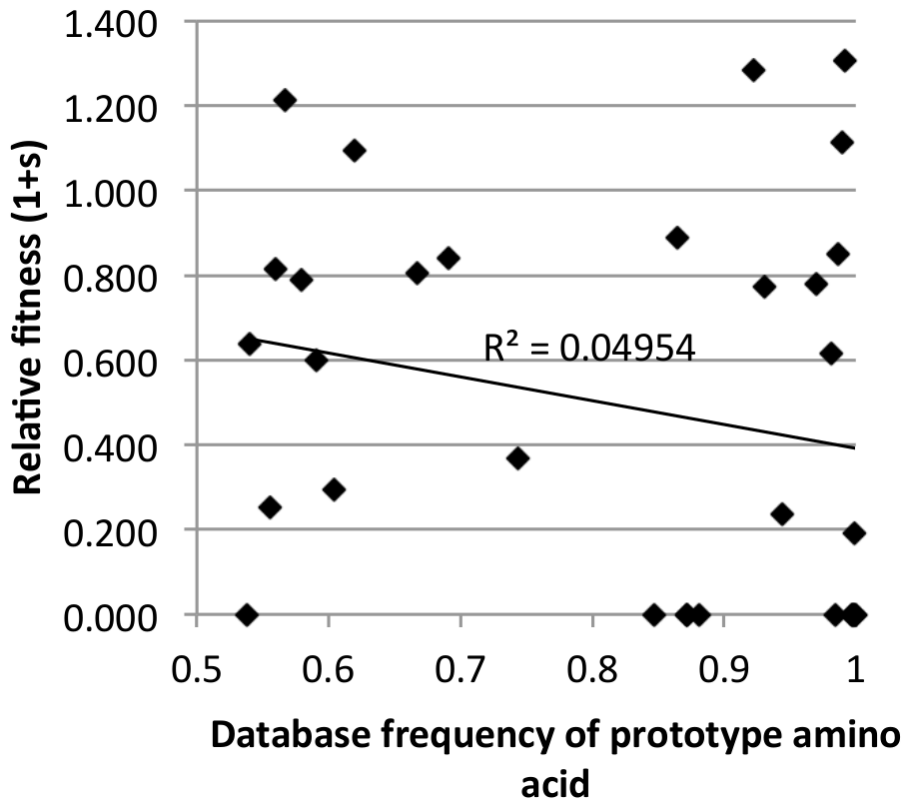
Relationship between sequence conservation and replication fitness. Relative fitness of all mutants evaluated as a function of database frequency of the amino acid found in the prototype COTM-CA sequence. Values shown are an average from two experiments, done in triplicate. The replication fitness of non-viable viruse is plotted as zero.

To examine the relationship between amino acid conservation and the mutational impact on viral fitness, the sites chosen for mutagenesis were differentially conserved, from a database frequency of 0.54 to 1.00 (Table 1). We found little direct relationship between the viral fitness and the database frequency of the prototype residue, with a non-significant Spearman’s correlation coefficient ρ = −0.27 (p = 0.136) and r^2^ = 0.05 (p = 0.788) (Figure 4). This observation also held true without the inclusion of non-viable mutants (data not shown).

### Mutations at Interface Sites were more Likely to be Deleterious and had Higher Fitness Costs than Mutations at Non-interface Sites

**Figure 5:**
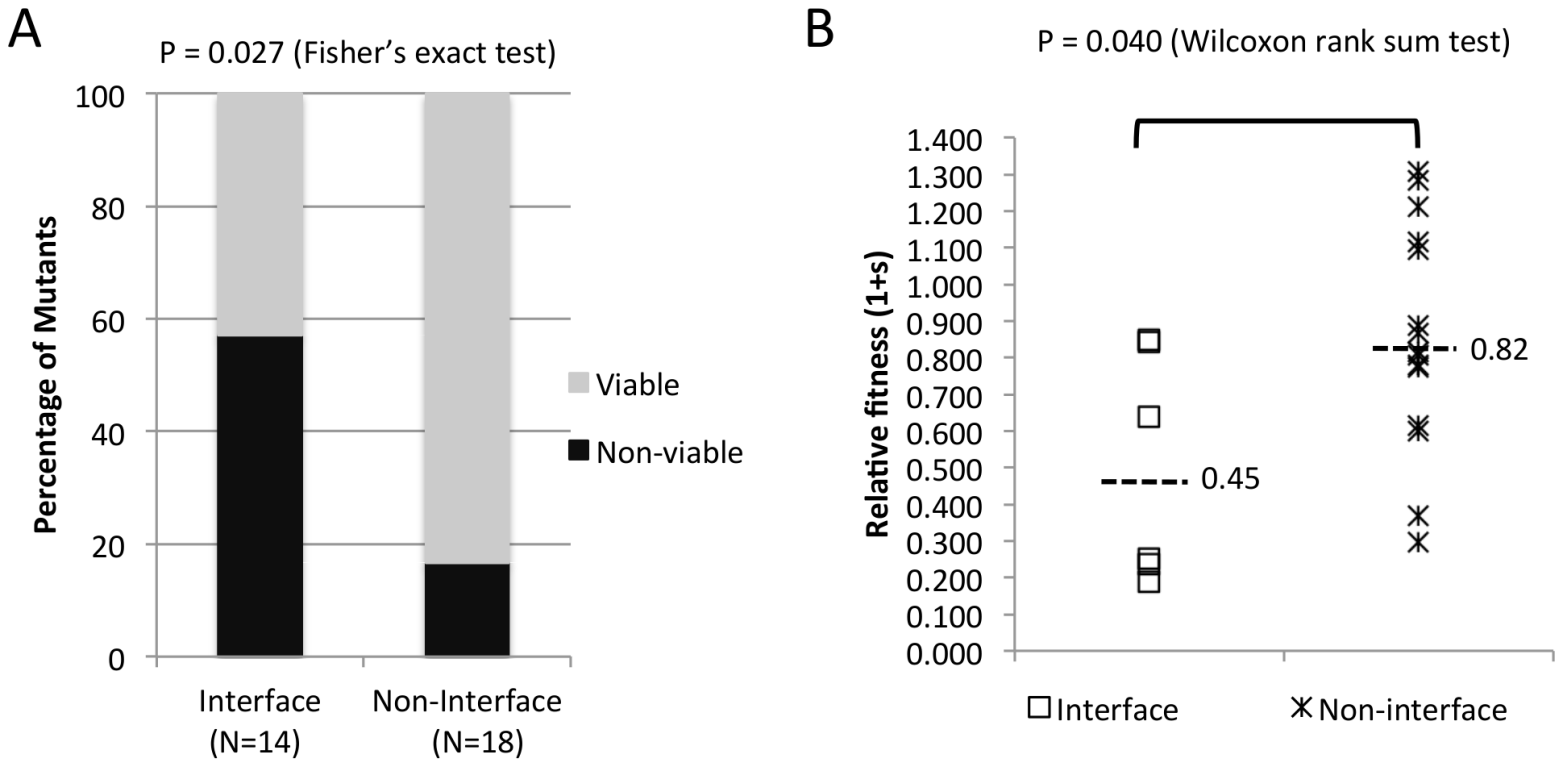
Viability and replication fitness of interface and non-interface mutants. **A**) Fraction of viable and non-viable mutants in each group. **B**) Relative fitness of viable mutants at interface and non-interface sites. Values shown are an average from two experiments done in triplicate.

**Figure 6:**
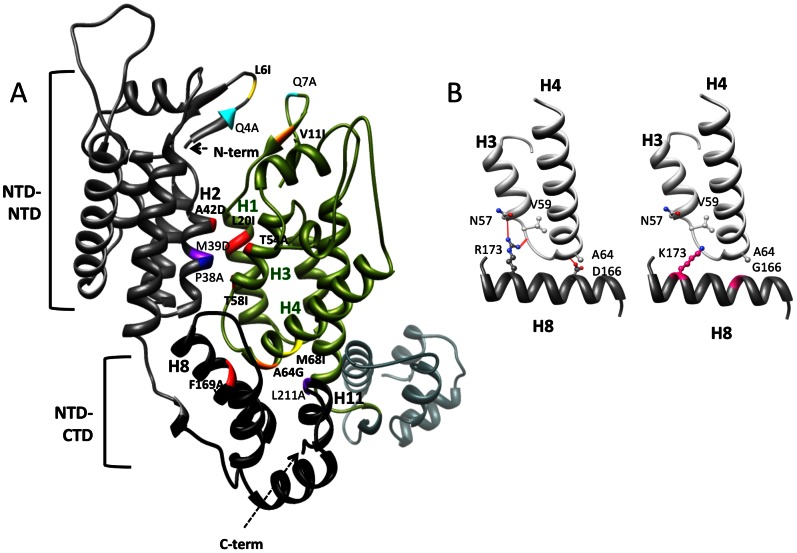
Structural localization of interface mutations. **A**) Two CA chains are shown as gray/black and green ribbons. The interface residues evaluated in this study are highlighted in bold and red-orange-yellow color. Other previously studied residues [7], [8] are highlighted in cyan-blue-purple color. The fitness impact of mutations is represented by the color shade ranging from small (yellow/cyan), moderate (orange/blue) to lethal (red/purple). **B**) Prototype residues participating in inter-domain helix capping hydrogen bonds, represented by orange lines, are shown on left. The mutations that resulted in loss of hydrogen bonds are modeled and highlighted on the right.

Mutations at interface sites were significantly more likely to be lethal, with 8/14 mutants resulting in non-viable viruses compared to 3/18 non-interface mutants (Fisher’s exact test p = 0.027) (Figure 5A and Table 1). In addition, mutations at interface sites had significantly greater negative impact on viral fitness among the viable mutant viruses studied (Wilcoxon rank sum test p = 0.040) (Figure 5B and Table 1). Among the fourteen interface mutations, seven were in NTD-NTD and NTD-CTD interfaces (Table 1). Mutations at either interface were not significantly different in the generation of non-viable (Fisher’s exact test p = 0.30) or less fit mutants (Student’s t-test p = 0.32). For the NTD-NTD interface, five mutations (L20I, A42D, T54M, T54A and T58I) were located in a small patch of hydrophobic contacts between helix 1, 2 and 3, and two mutations (L6I and V11I) were situated in the beta strand at the beginning of the NTD. All five mutations in helix bundles resulted in non-viable viruses. On the other hand, both mutations in the beta strand resulted in viable viruses with L6I having a small (mean 1+s = 0.85) and V11I having a substantial (1+s = 0.24) negative impact on viral fitness (Figure 6A). For the NTD-CTD interface, four mutations (A64G, M68I, F169Y and F169A) were situated in a cluster of hydrophobic contacts between helix 4, 8 and 11 and had various impacts on viral replication, from minor decreases to lethality (Figure 6A). The other three mutations were at sites forming inter-domain helix-capping motifs for helix 3, 4 and 8. Two of these, R173K and D166G, resulted in a loss of side-chain atoms required for hydrogen bonding (Figure 6B), and yielded non-viable mutants (Figure 2), while the other, D71E, also had a substantial negative impact on viral fitness (Figure 3).

## Discussion

We assessed the replication fitness cost of thirty-two single amino acid substitutions in the HIV-1 CA of the HIV-1 M group Center-of-Tree sequence. The COTM-CA sequence was derived from all group M viruses to represent the central point of viral sequence diversity. As such, the COTM-CA sequence is not identical to any natural isolate of HIV-1 but rather a coalescence of prototype amino acid from all subtypes, especially the two best studied, B and C. In pairwise competition assays, the chimeric NL4-3 COTM-CA virus showed slower growth kinetics than the prototype NL4-3, a highly cell culture adapted subtype B virus. The lower relative fitness of the recombinant NL4-3 COTM-CA virus may be due to less optimal interactions between COTM-CA and other NL4-3 viral proteins. Alternatively, by combining multiclade prototype residues, we might have disrupted subtype specific co-evolving residual pairs, and in doing so negatively impacted the CA structure and/or function and hence viral replication fitness. Arguing against this possibility, however, the COTM-CA sequence retains almost all subtype specific co-evolving residues that have been reported previously [28]. The only exception is at the residue 41 and 120 pairing, in which the COTM-CA sequence contains serine at both position 41 and 120 of CA. The mutation S41T, which recovers the naturally observed co-variation threonine at 41 and serine at 120 [28] (Table S4), did not show a significant impact on the prototype COTM virus replication fitness (data not shown). Thus, despite slower growth kinetics than the highly cell-line adapted NL4-3 virus, the COTM-CA virus was infectious and applicable as the prototype virus for studying the effect of mutations within CA on viral fitness.

From thirty-two single amino acid substitutions introduced into the COTM-CA virus, eleven were lethal and seventeen had fitness costs, while four appeared to yield faster replicating capacities. The majority of the lethal mutations were located in the interface between CA subunits within a capsid hexamer. X-ray crystallographic studies identified two interfaces between capsid hexamer subunits: NTD-NTD and NTD-CTD. Both interfaces consist of a small cluster of hydrophobic contacts and a more extensive network of water mediated hydrogen bonds [5]. In this study, the hydrophobic contacts at NTD-NTD were highly sensitive to change and even conservative amino acid substitutions were found to be deleterious. On the other hand, mutations at interface residues at the beginning of the NTD, which are outside of the hydrophobic clusters, showed less impact on viral replication. The same cluster of hydrophobic contacts at NTD-NTD is also observed in the x-ray crystal structure of HIV-1 capsid pentamer [29]. Previous studies showed that alanine mutagenesis and substitution of hydrophilic for hydrophobic residues in this otherwise hydrophobic cluster drastically reduced viral infectivity and, in some cases, altered mature capsid morphology but not particle production [6], [7], [9]. Taken together, this suggests that stabilization of capsid hexameric and pentameric subunits through this NTD-NTD hydrophobic cluster is crucial for the capsid function and, hence, essential for viral replication.

Mutations at hydrophobic residues at the NTD-CTD interface also negatively affected viral replication. However, these effects seemed to be smaller, as only the F169A mutation was lethal. The crucial interactions within the NTD-CTD interface were the inter-domain helix capping hydrogen bonds, which have been speculated to act as pivotal points for allowing one domain to move relative to another [5]. A similar structural motif was observed in the capsid hexamer of Rous sarcoma virus [30]. These pivotal points were suggested to be responsible for generating the continuously curved surfaces observed across retroviral capsids [5]. Five inter-domain helix-capping interactions were observed in the crystal structure of the HIV-1 capsid hexamer, three of which were consistently observed while the other two were found only in some protomers [5]. Among the three consistently observed interactions, R173 and D166 were suggested to be the most critical amino acids, based on the HIV-1 and RSV capsid structures, respectively [5], [30]. Our results support these observations, as the mutations that disrupted these hydrogen bonds, R173K and D166G, were lethal.

Non-interface mutations included in this study were significantly less likely to be lethal (Fisher’s exact test p = 0.027) (Figure 5A) and showed less negative effects on viral replication (Wilcoxon rank sum test p = 0.040). Nevertheless, three mutations (H12Y, F161S and T200S) resulted in non-infectious mutants and two others (E128D and I124V) had substantial negative impacts on viral fitness, suggesting that these residues are important for CA functions other than the mature capsid assembly. On the other hand, four mutations (T48A, G225S, E98D and E45D) had positive effects on viral fitness. It remains to be established how these mutations affect CA structure and function.

In addition to exploring the association between structural localization and relative fitness, we examined the relationship between relative fitness and sequence conservation and found a non-significant trend between these two properties. Although the number of sites was limited to thirty, which accounts for about one-seventh of CA, our observation should not be particularly affected by sampling. The lack of direct relationship between sequence conservation and the relative fitness was surprising but not entirely unexpected [31]. Sequence conservation is influenced by evolutionary constraints, such as structural and functional requirements, and selective pressures, such as host immune pressure, as well as random genetic drift. The results presented here reflect the immediate impact of mutations on viral replication in a permissive T cell line, which would reflect CA structural and functional constraints but not host-specific selective pressures. In addition, our study focused on characterizing the effect of single mutations and hence did not account for possible occurrences of compensatory mutations, which could be part of the reason why some of the lethal mutations we observed, T54M, T58I and T200S, were found in noticeable fractions (∼10%) of the HIVDB. Interestingly, these mutations were more frequently observed in non-B and non-C subtypes (Table 1 and Table S5). It is possible that their hypothetical compensatory mutations are missing from our recombinant COTM-CA virus. Besides these three mutations, the other eight lethal mutations were rare and observed in ∼1% or lower fraction of the HIVDB across all group M viruses (Table 1 and Table S5). To assess the influence of genetic background on the fitness effect, we compared results between this study and the fitness results from a study done in subtype B virus ([31], including 19 overlapping mutations with 6 at capsid hexamerization interface sites. We found a significant positive relationship between fitness effects in the two studies (r^2^ = 0.544, p = 0.0003; Spearman’s Rho = 0.774, p = 9.605E–05) (Figure S3). Although our results may be affected by the limited capability of the *in vitro* fitness assay to recapitulate evolutionary forces *in vivo*, it indicates that extrapolating the direct impact of mutations on viral replication capability from the sequence conservation alone can be inaccurate and that incorporation of structural and functional information is essential.

Our observation that mutations at interface sites were likely to have a large negative impact on viral replication is in agreement with previous studies [7], [8] and indicate the importance of capsid hexamer stabilization on viral replication. However, certain side-chain interactions, such as NTD-NTD hydrophobic contacts and NTD-CTD helix capping hydrogen bonds, were found to be more important than others in maintaining capsid hexamer structures. As such they represent vulnerable targets that can be exploited in drug development and also vaccine designs that seek to target epitopes encompassing conserved elements critical to viral function [15], [16], [32], [33].

## Supporting Information

Figure S1 **Viral ratio in pair-wise growth competition assay.** Values shown are the average taken from one representative experiment done in triplicate. The error bar represents the standard deviation between the triplicates
(TIF)

Figure S2 **CA production in transfected 293T culture supernatant.**
(TIF)

Figure S3 **Positive relationship between fitness effects in two strains.**
(TIF)

Table S1 **Primers used to create new restriction sites and CA mutations in pNL4-3 plasmid.**
(DOCX)

Table S2 **Distribution of HIV-1 subtypes in the dataset used to calculate amino acid sequence conservation and derive the COTM sequence.**
(DOCX)

Table S3 **Database frequency of the consensus amino acid of group M HIV-1 CA.**
(DOCX)

Table S4 **Subtype B, subtype C consensus and COTM-CA amino acid at the co-evolving residual pair.** Taken from [28].
(DOCX)

Table S5 **Amino acid database frequency of subtype B, subtype C and other group M sequences.** Base on database frequency, the 30 amino acid sites included in this study can be categorized into three groups (identified by superscripts in “Mutation” column). The first group contains 18 sites, whose mutation pattern was conserved among group M subtypes, i.e., the most frequent and the second most frequent amino acid were the same in both Subtypes B and C. The second group consists of 8 sites, in which the most frequent and the second most frequent residue found in subtype B and subtype C were opposite. For example, at site 71, the most frequent and the second most frequent amino acid was glutamic acid (0.93) and aspartic acid (0.05), respectively, in subtype B sequences. But in subtype C sequences, it was aspartic acid (0.98) and then glutamic acid (0.01). The third group contains 4 mutations, whose mutation pattern in subtype B and subtype C are similar to each other but different from that of other group M subtypes.
(DOCX)
