# Supplementary material for: Fitness Costs of Mutations at the HIV-1 Capsid Hexamerization Interface
Source: PLoS One. 2013 Jun 13;8(6):e66065. doi: 10.1371/journal.pone.0066065 (PMC3681919; doi:10.1371/journal.pone.0066065)
Supplement: Table S1 — Primers used to create new restriction sites and CA mutations in pNL4-3 plasmid. (DOCX) [file pone.0066065.s004.docx]

**Table S1. Primers used to create new restriction sites and CA mutations in pNL4-3 plasmid.**

| **CA mutant/restriction sites** | **5'-3' (forward) sequence (mutations in bold)** |
| --- | --- |
| *sfi*I | CAAGCAGAAG**G**C**C**CAGCAGGC**C**GCGGCCGACA |
| *bst*EII | CGAGGCGATGAGCCA**G**GTG**A**C**C**AACACGACGATCATG |
| L6I | CCGATCGTGCAGAAC**A**TCCAGGGACAGATGG |
| V11I | TCCAGGGACAGATG**A**TCCACCAGGCCATC |
| H12Y | CAGGGACAGATGGTC**T**A**T**CAGGCCATCTCCCCA |
| I15L | CACCAGGCC**C**TCTCCCCACGGACGCTTAAC |
| L20I | CTCCCCACGGACG**A**TTAACGCGTGGGT |
| I27V | TAACGCGTGGGTCAAAGTA**G**TCGAGGAGAAGGC |
| A42D | TCCCCATGTTCTCGG**AT**CTTTCCGAGGGAGCC |
| S44A | CATGTTCTCGGCACTT**G**CCGAGGGAGCCAC |
| E45D | GTTCTCGGCACTTTCCGA**T**GGAGCCACCC |
| T48A | CCGAGGGAGCC**G**CCCCGCAGGAC |
| T54A | CCGCAGGACCTGAAC**G**CGATGTTGAACACCG |
| T54M | CCGCAGGACCTGAACA**T**GATGTTGAACACCGTC |
| T58I | CTGAACACGATGTTGAACA**T**CGTCGGCGGG |
| A64G | CGGGCACCAGG**G**GGCCATGCAGA |
| M68I | GGCGGCCATGCAGAT**A**CTTAAGGACACCATC |
| D71E | CAGATGCTTAAGGA**G**ACCATCAACGAGGAG |
| E98D | GGGCCAGATGAGAGA**T**CCGCGGGG |
| T110N | CGGGAACCACCAGCA**A**CTTGCAGGAGCAAAT |
| I124V | TGACTTCGAACCCGCCA**G**TCCCGGTCG |
| E128D | CAATCCCGGTCGGGGA**T**ATCTACAAGAGATGGA |
| V148T | GGATGTACAGCCCT**AC**CAGCATCCTGGAC |
| R154K | CAGCATCCTGGACATC**AA**ACAGGGACCGAAGGAG |
| F161S | GGGACCGAAGGAGCCG**AG**CAGAGACTACGTAGAC |
| D166G | GTTCAGAGACTACGTAG**G**CCGGTTCTTCAAGACTC |
| F169Y | TACGTAGACCGGTTCT**AT**AAGACTCTCCGGGCGG |
| F169A | CTACGTAGACCGGTTC**GC**CAAGACTCTCCGGGCG |
| R173K | GTTCTTCAAGACTCTC**AA**GGCGGAGCAGGCGACG |
| T200S | CCCGGACTGCAAGA**G**CATCCTGAAGGCTC |
| K203R | GACTGCAAGACCATCCTGA**G**GGCTCTCGGC |
| T116S | TGGAAGAGATGATG**T**CGGCGTGCCAGGG |
| G225S | AGGGAGTCGGGGGACCC**A**GCCACAAGGCG |
| V230I | CACAAGGCGCGG**A**TCTTGGCCGAGG |
