# Supplementary material for: Fitness Costs of Mutations at the HIV-1 Capsid Hexamerization Interface
Source: PLoS One. 2013 Jun 13;8(6):e66065. doi: 10.1371/journal.pone.0066065 (PMC3681919; doi:10.1371/journal.pone.0066065)
Supplement: Table S2 — Distribution of HIV-1 subtypes in the dataset used to calculate amino acid sequence conservation and derive the COTM sequence. (DOCX) [file pone.0066065.s005.docx]

**Table S2. Distribution of HIV-1 subtypes in the dataset used to calculate amino acid sequence conservation and derive the COTM sequence.**

| **HIV-1 Subtypes in Group M** | **Number of sequences** | **Percentage** |
| --- | --- | --- |
| Subtype A | 39 | 3.83 |
| Subtype B | 411 | 40.33 |
| Subtype C | 408 | 40.04 |
| Subtype D | 18 | 1.77 |
| Subtype F | 24 | 2.36 |
| Subtype G | 8 | 0.79 |
| Subtype H | 2 | 0.20 |
| Subtype J | 1 | 0.10 |
| Subtype K | 2 | 0.20 |
| CRFs | 106 | 10.40 |
| **Total** | **1019** | **100.00** |
