# Supplementary material for: Fitness Costs of Mutations at the HIV-1 Capsid Hexamerization Interface
Source: PLoS One. 2013 Jun 13;8(6):e66065. doi: 10.1371/journal.pone.0066065 (PMC3681919; doi:10.1371/journal.pone.0066065)
Supplement: Table S3 — Database frequency of the consensus amino acid of group M HIV-1 CA. (DOCX) [file pone.0066065.s006.docx]

**Table S3. Database frequency of the consensus amino acid of group M HIV-1 CA**

| **Sites (# of sites)** | **Average database frequency of consensus residue** |
| --- | --- |
| All CA sites (231) | 0.941 |
| All interface sites (48) | 0.936 |
| Interface sites in this study (12) | 0.872 |
| Non-interface sites in this study (21) | 0.810 |
