# Supplementary material for: Fitness Costs of Mutations at the HIV-1 Capsid Hexamerization Interface
Source: PLoS One. 2013 Jun 13;8(6):e66065. doi: 10.1371/journal.pone.0066065 (PMC3681919; doi:10.1371/journal.pone.0066065)
Supplement: Table S4 — Subtype B, subtype C consensus and COTM-CA amino acid at the co-evolving residual pair. Taken from [28]. (DOCX) [file pone.0066065.s007.docx]

**Table S4. Subtype B, subtype C consensus and COTM-CA amino acid at the co-evolving residual pair.** Taken from [28].

| **Co-evolving residues** | **Site^a^** | **B consensus** | **C consensus** | **COTM-CA** |
| --- | --- | --- | --- | --- |
| 27-148 | 27 | V | **I** | **I** |
|  | 148 | T | **V** | **V** |
| 41-120* | 41 | **S** | T | **S** |
|  | 120 | N | **S** | **S** |
| 71-169 | 71 | E | **D** | **D** |
|  | 169 | Y | **F** | **F** |
| 116-124 | 116 | **G** | A | **G** |
|  | 124 | **I** | V | **I** |
| 148-169 | 148 | T | **V** | **V** |
|  | 169 | Y | **F** | **F** |
| 187-208 | 187 | E | **D** | **D** |
|  | 208 | A | **G** | **G** |

^a^ Site numbering correspond to Gag protein of HIV-1 HXB2 strain

* Indicates subtype specific co-evolving residual pair that is not maintained in our COTM-CA sequence
