# Supplementary material for: Fitness Costs of Mutations at the HIV-1 Capsid Hexamerization Interface
Source: PLoS One. 2013 Jun 13;8(6):e66065. doi: 10.1371/journal.pone.0066065 (PMC3681919; doi:10.1371/journal.pone.0066065)
Supplement: Table S5 — Amino acid database frequency of subtype B, subtype C and other group M sequences. Base on database frequency, the 30 amino acid sites included in this study can be categorized into three groups (identified by superscripts in “Mutation” column). The first group contains 18 sites, whose mutation pattern was conserved among group M subtypes, i.e., the most frequent and the second most frequent amino acid were the same in both Subtypes B and C. The second group consists of 8 sites, in which the most frequent and the second most frequent residue found in subtype B and subtype C were opposite. For example, at site 71, the most frequent and the second most frequent amino acid was glutamic acid (0.93) and aspartic acid (0.05), respectively, in subtype B sequences. But in subtype C sequences, it was aspartic acid (0.98) and then glutamic acid (0.01). The third group contains 4 mutations, whose mutation pattern in subtype B and subtype C are similar to each other but different from that of other group M subtypes. (DOCX) [file pone.0066065.s008.docx]

**Table S5. Amino acid database frequency of subtype B, subtype C and other group M sequences.** Base on database frequency, the 30 amino acid sites included in this study can be categorized into three groups (identified by superscripts in “Mutation” column). The first group contains 18 sites, whose mutation pattern was conserved among group M subtypes, i.e., the most frequent and the second most frequent amino acid were the same in both Subtypes B and C. The second group consists of 8 sites, in which the most frequent and the second most frequent residue found in subtype B and subtype C were opposite. For example, at site 71, the most frequent and the second most frequent amino acid was glutamic acid (0.93) and aspartic acid (0.05), respectively, in subtype B sequences. But in subtype C sequences, it was aspartic acid (0.98) and then glutamic acid (0.01). The third group contains 4 mutations, whose mutation pattern in subtype B and subtype C are similar to each other but different from that of other group M subtypes.

|  | **Mutation** | **Most frequent amino acid frequency** | | | | **Mutant amino acid frequency** | | | |
| --- | --- | --- | --- | --- | --- | --- | --- | --- | --- |
|  |  | **Group M (n=1019)** | **Subtype B (n=411)** | **Subtype C (n=408)** | **Others (n=200)** | **Group M (n=1019)** | **Subtype B (n=411)** | **Subtype C (n=408)** | **Others (n=200)** |
| NTD-NTD | L6I^3^ | 0.687 | 0.713 | 0.860 | 0.280 | 0.066 | 0.114 | 0.039 | 0.021 |
|  | V11I^1^ | 0.939 | 0.976 | 0.973 | 0.795 | 0.037 | 0.015 | 0.012 | 0.135 |
|  | L20I^1^ | 1.000 | 1.000 | 1.000 | 1.000 | 0.000 | 0.000 | 0.000 | 0.000 |
|  | A42D^1^ | 1.000 | 1.000 | 1.000 | 1.000 | 0.000 | 0.000 | 0.000 | 0.000 |
|  | T54M^3^ | 0.867 | 0.971 | 0.953 | 0.475 | 0.096 | 0.002 | 0.005 | 0.475 |
|  | T54A^3^ | 0.867 | 0.971 | 0.953 | 0.475 | 0.005 | 0.002 | 0.005 | 0.010 |
|  | T58I^3^ | 0.876 | 0.978 | 0.968 | 0.480 | 0.103 | 0.002 | 0.010 | 0.500 |
| NTD-CTD | A64G^1^ | 0.999 | 1.000 | 1.000 | 0.995 | 0.001 | 0.000 | 0.000 | 0.005 |
|  | M68I^1^ | 0.981 | 0.968 | 0.990 | 0.990 | 0.013 | 0.024 | 0.002 | 0.010 |
|  | D71E^2^ | 0.554 | 0.054 | 0.983 | 0.705 | 0.439 | 0.934 | 0.012 | 0.290 |
|  | D166G^1^ | 0.998 | 1.000 | 0.995 | 1.000 | 0.002 | 0.000 | 0.005 | 0.000 |
|  | F169Y^2^ | 0.538 | 0.000 | 0.995 | 0.710 | 0.456 | 0.993 | 0.000 | 0.285 |
|  | F169A^2^ | 0.538 | 0.000 | 0.995 | 0.710 | 0.000 | 0.000 | 0.000 | 0.000 |
|  | R173K^1^ | 0.999 | 1.000 | 0.998 | 1.000 | 0.001 | 0.000 | 0.002 | 0.000 |
| Non-interface | H12Y^1^ | 0.984 | 1.000 | 0.993 | 0.935 | 0.013 | 0.000 | 0.002 | 0.060 |
|  | I15L^3^ | 0.617 | 0.720 | 0.650 | 0.340 | 0.320 | 0.253 | 0.321 | 0.455 |
|  | I27V^2^ | 0.575 | 0.251 | 0.880 | 0.620 | 0.419 | 0.742 | 0.113 | 0.380 |
|  | S44A^1^ | 0.965 | 0.925 | 0.990 | 0.995 | 0.029 | 0.068 | 0.002 | 0.005 |
|  | E45D^1^ | 0.987 | 0.990 | 0.983 | 0.990 | 0.008 | 0.002 | 0.012 | 0.010 |
|  | T48A^1^ | 0.984 | 0.988 | 0.983 | 0.980 | 0.006 | 0.000 | 0.007 | 0.015 |
|  | E98D^1^ | 0.918 | 0.912 | 0.914 | 0.935 | 0.074 | 0.080 | 0.076 | 0.055 |
|  | T110N^1^ | 0.861 | 0.876 | 0.821 | 0.910 | 0.118 | 0.110 | 0.145 | 0.080 |
|  | I124V^1^ | 0.739 | 0.929 | 0.556 | 0.720 | 0.246 | 0.056 | 0.422 | 0.280 |
|  | E128D^2^ | 0.601 | 0.895 | 0.302 | 0.605 | 0.390 | 0.090 | 0.689 | 0.395 |
|  | V148T^2^ | 0.663 | 0.238 | 0.968 | 0.915 | 0.274 | 0.630 | 0.015 | 0.070 |
|  | R154K^2^ | 0.555 | 0.698 | 0.373 | 0.635 | 0.438 | 0.294 | 0.618 | 0.365 |
|  | F161S^1^ | 0.993 | 0.993 | 0.993 | 0.995 | 0.002 | 0.000 | 0.003 | 0.005 |
|  | T200S^1^ | 0.842 | 0.961 | 0.880 | 0.520 | 0.091 | 0.005 | 0.025 | 0.405 |
|  | K203R^2^ | 0.588 | 0.983 | 0.196 | 0.575 | 0.406 | 0.010 | 0.799 | 0.420 |
|  | T216S^1^ | 0.976 | 0.964 | 0.985 | 0.985 | 0.009 | 0.017 | 0.003 | 0.005 |
|  | G225S^2^ | 0.562 | 0.679 | 0.490 | 0.470 | 0.416 | 0.287 | 0.500 | 0.510 |
|  | V230I^1^ | 0.925 | 0.869 | 0.971 | 0.950 | 0.066 | 0.119 | 0.020 | 0.050 |
| Co-evolving residues | S41T^2^ | 0.474 | 0.742 | 0.037 | 0.815 | 0.482 | 0.168 | 0.949 | 0.175 |
|  | N120S^2^ | 0.336 | 0.535 | 0.201 | 0.200 | 0.432 | 0.248 | 0.539 | 0.590 |
|  | G116A^2^ | 0.391 | 0.681 | 0.032 | 0.525 | 0.443 | 0.226 | 0.789 | 0.180 |
|  | D187E^2^ | 0.394 | 0.002 | 0.787 | 0.395 | 0.594 | 0.985 | 0.203 | 0.585 |
|  | G208A^2^ | 0.619 | 0.236 | 0.865 | 0.905 | 0.365 | 0.752 | 0.123 | 0.065 |

^1^ Amino acid site whose frequency patterns are similar in all group M sequences

^2^ Amino acid site whose frequency pattern in subtype B is the opposite of subtype C. The pattern in other group M sequences is either similar to subtype B or subtype C.

^3^ Amino acid site, whose frequency pattern in subtype B is similar to subtype C, but different from other group M sequences
